# The Integrated Treatment of Psychotherapy, Body Therapy, Sociotherapy, and Psychopharmacotherapy in the Bio-Psycho-Social Model with Psychological Prevalence: Three Clinical Cases

**DOI:** 10.1192/j.eurpsy.2026.10925

**Published:** 2026-07-31

**Authors:** M. M. Rega, F. Del Prete, E. Ammendola

**Affiliations:** Società Italiana di Psicoterapia Integrata, Casoria (NA), Italy

## Abstract

**Introduction:**

This paper presents an analysis of three clinical cases treated with two versions of the bio-psycho-social model:

1. Model with Biological Prevalence: The treatment of choice is pharmacological, complemented by psychotherapy and supportive sociotherapy. The aim is to reduce the severity of symptoms, but this results in a reduction of the capabilities of the patient.
2. Model with Psychological Prevalence: The treatment of choice is psychotherapy, with psychopharmacotherapy as a supportive measure. The aim is to increase awareness of maladaptive relational rules that generate symptoms to facilitate the decision to modify them.

**Objectives:**

The objective of this work is to demonstrate that the bio-psycho-social model with psychological prevalence facilitates the understanding of the psychotic patient, leading to increased treatment efficiency.

**Methods:**

In the integrated treatment approach, the method applied is a unique intervention model that seeks to identify the stable rules of functioning of the patient and their family. This model guides interventions, which are focused on awareness and decision-making, across all settings of the care community:

- Individual, family, group psychotherapy and body therapy
- Sociotherapy: Workshops with four levels of increasing autonomy
- Psychopharmacotherapy: To reduce the pain of awareness and decision-making
- Supervision: To develop common guidelines and integration of interventions

**Results:**

The results achieved from this approach include:

- Symptom reduction
- Reduction in voluntary and compulsory medical treatments
- Reduction in psychopharmacotherapy (dosage and costs)
- Affective autonomy and work-related autonomy

**Conclusions:**

In conclusion, we highlight the opportunity to develop the different forms of the bio-psycho-social model with psychological prevalence.

**Disclosure of Interest:**

None Declared

